# Comment on 'The impact of vitamin D pathway genetic variation and circulating 25-hydroxyvitamin D on cancer outcome: systematic review and meta-analysis'

**DOI:** 10.1038/bjc.2017.184

**Published:** 2017-06-22

**Authors:** Alain Braillon

**Affiliations:** 1 Department of Medicine, University Hospital, 80000 Amiens, France

**Sir**,

Vaughan-Shaw *et al* (2017) claimed that 25(OH)D concentration is associated with better cancer outcome, and the observed association of functional variants in vitamin D pathway genes with outcome supports a causal link. This deserves a comment.

First, using arbitrary cut-points to derive subgroups for 25(OH)D is not appropriate when there is a continuous distribution of the values with no obvious modal values.

Second, no adjustments for main confounding clinical variables were performed. For smoking it would have been a catch-22: (a) smokers have lower 25(OH)D (Tønnesen *et al*, 2016); (b) 25(OH)D is associated with higher risk of tobacco-related cancers (Afzal *et al*, 2013). Similarly, alcohol consumption, obesity, overweight, insulin resistance, type 2 diabetes have an impact on vitamin D status (Palaniswamy *et al*, 2017).

Long ago, in 1998, a prospective survey (NHANES III) investigated 25(OH)D levels with mortality, accounting for age, sex, ethnicity, diabetes, current smoking, body mass index, physical activity, supplementation, season and so on (Melamed *et al*, 2008). Personalised medicine is first about phenotyping not genotyping!
